# ADAPTIVE GARDENING PRACTICES AMONG OLDER AFRICAN AMERICANS IN DETROIT

**DOI:** 10.1093/geroni/igz038.1901

**Published:** 2019-11-08

**Authors:** Jessica C Robbins, Kimberly Seibel

**Affiliations:** Wayne State University, Detroit, Michigan, United States

## Abstract

It is well established that gardening can promote physical, social, and emotional wellbeing for many older adults in varied circumstances (Milligan, Gatrell, and Bingley 2004; Nicklett, Anderson, and Yen 2016; Wang and MacMillan 2013). In post-industrial cities formed by historical and ongoing processes of structural inequality such as Detroit, Michigan, gardening is beneficial for residents in terms of health, economic activity, community-building, and city beautification (Lawson 2005; Pitt 2014; Pothukuchi 2015; White 2011). However, research has less frequently investigated how gardening can promote wellbeing for older adults living in contexts of urban structural inequality. This poster addresses this gap by exploring how older African American gardeners in Detroit adapt their gardening practices to changing physical abilities and capacities. Drawing on ethnographic research conducted during one gardening season (March-October 2017) with older African Americans in Detroit (n= 27), we employ a selective-optimization-with-compensation framework (Baltes and Baltes 1990) to understand the modifications that older Detroiters make in their gardening practices as they age. Findings demonstrate that older African Americans in Detroit engage in gardening in flexible, creative ways that accommodate new physical limitations, while also connecting to changes occurring in the city of Detroit. This study thus has implications for further understanding how gardening can benefit older adults, and how older adults can contribute vitality to contexts of structural inequality.

